# Effects of Shade Treatments on Photosynthetic Characteristics, Chloroplast Ultrastructure, and Physiology of *Anoectochilus roxburghii*

**DOI:** 10.1371/journal.pone.0085996

**Published:** 2014-02-07

**Authors:** Qingsong Shao, Hongzhen Wang, Haipeng Guo, Aicun Zhou, Yuqiu Huang, Yulu Sun, Mingyan Li

**Affiliations:** 1 The Nurturing Station for the State Key Laboratory of Subtropical Silviculture, Zhejiang A & F University, Hangzhou, China; 2 Zhejiang University, Hangzhou, China; 3 Zhejiang Shouxiangu Rare Botanical Institute, Wuyi, China; Louisiana State University and A & M College, United States of America

## Abstract

*Anoectochilus roxburghii* was grown under different shade treatments–50%, 30%, 20%, and 5% of natural irradiance–to evaluate its photosynthetic characteristics, chloroplast ultrastructure, and physiology. The highest net photosynthetic rates and stomatal conductance were observed under 30% irradiance, followed in descending order by 20%, 5%, and 50% treatments. As irradiance decreased from 50% to 30%, electron transport rate and photochemical quenching increased, while non-photochemical quenching indexes declined. Reductions in irradiance significantly increased Chl a and Chl b contents and decreased Chl a/b ratios. Chloroplast ultrastructure generally displayed the best development in leaves subjected to 30% irradiance. Under 50% irradiance, leaf protein content remained relatively stable during the first 20 days of treatment, and then increased rapidly. The highest peroxidase and superoxide dismutase levels, and the lowest catalase activities, were observed in plants subjected to the 50% irradiance treatment. Soluble sugar and malondialdehyde contents were positively correlated with irradiance levels. Modulation of chloroplast development, accomplished by increasing the number of thylakoids and grana containing photosynthetic pigments, is an important shade tolerance mechanism in *A. roxburghii*.

## Introduction

*Anoectochilus roxburghii*, a member of the Orchidaceae, is a valued plant species in many Asian countries, where it is used for ornamental, culinary, and medicinal purposes. Because of its unique medicinal properties, such as its notable curative effects of clearing heat and cooling blood, eliminating dampness, and detoxification, *A. roxburghii* has been called “the king of medicine” [1], [2]. Recent research has demonstrated that the entire plant possesses medicinal properties, such as antioxidant, anti-inflammatory, and antitumor activities [3], [4]. *A. roxburghii* has been traditionally harvested mainly from wild populations. The species has become endangered, however, as a result of human overexploitation coupled with its specific environmental growth requirements. As a consequence, artificial cultivation is beginning to be carried out in various locations in China.

Light has long been known to be the most important factor influencing plant growth, with changes in irradiance having impacts on plant growth, morphology, and anatomy, various aspects of physiology and cellular biochemistry, and, ultimately, flowering time and plant productivity [5]–[7]. In the light reactions of photosynthesis, light energy is used to produce ATP and NADPH, which are then used for carbon fixation to carbohydrates and production of oxygen during the light-independent phase. Shading effects are not just about the plants’ growth and development, but through which, it also has a major impact on plant photosynthesis. Normal plant growth needs optimal light irradiance because excessively high and low irradiances would result in photoinhibition and light deficiency respectively, and therein the growth of plant was restricted severely. Under high irradiance conditions, photoinhibition takes place: the photosynthetic apparatus absorbs excessive light energy, resulting in the inactivation or impairment of the chlorophyll-containing reaction centers of chloroplasts and consequent depression of photosynthetic activity [8], [9]. In contrast, under low irradiance conditions, insufficient ATP is produced to allow for carbon fixation and carbohydrate biosynthesis. This leads to a reduction in plant growth.

Chloroplasts are the sole organelles of photosynthesis. Although many authors have reported a close relationship among photosynthesis, chlorophyll content, and chlorophyll fluorescence in different species under shading conditions, little work has been performed on the association between photosynthesis-related parameters and chloroplast ultrastructure and physiology [10]. A comprehensive understanding of changes in chloroplast ultrastructure and physiology during leaf development under different irradiance conditions is needed.

The objectives of the present study were to quantify the influences of different shading levels on photosynthetic characteristics, chloroplast ultrastructure, and physiology of *A. roxburghii*, to determine optimum light intensity for plant growth, and to consequently expand our understanding of its shade-tolerance abilities and mechanisms. In addition, we wished to establish a sound foundation for improving the cultivation and breeding of this important plant species.

## Materials and Methods

### Plant Materials and Growth Conditions

**Figure 1:**
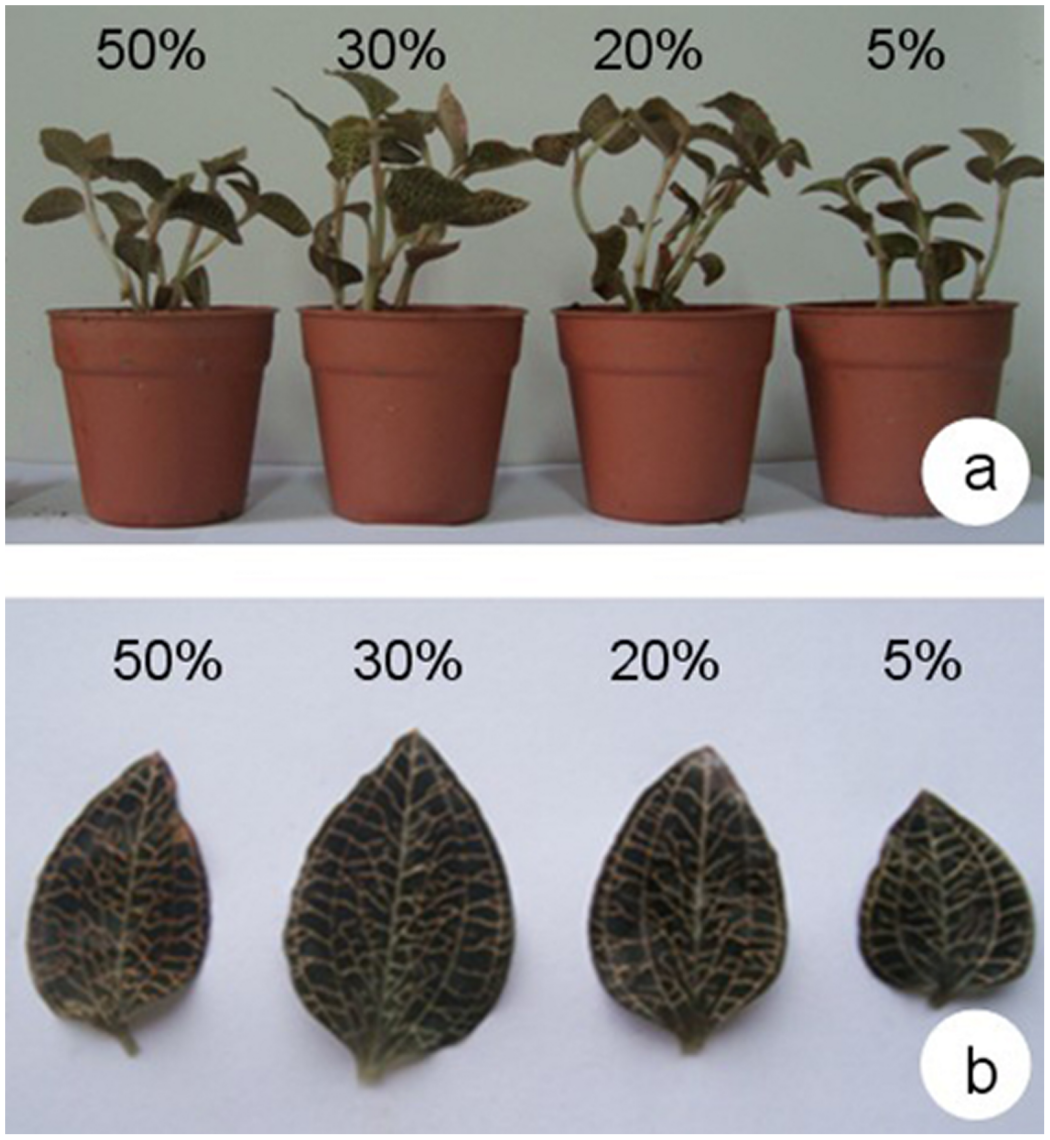
The appearance of whole plants (a) and leaves (b) exposed to 40 d of various levels of shading.

**Figure 2:**
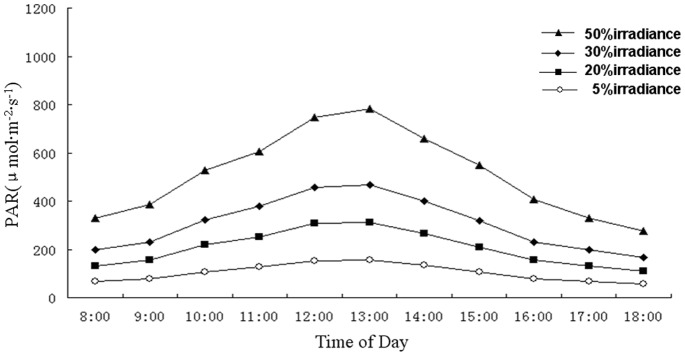
Curves of diurnal variation of photosynthetically active radiation (PAR) under 50%, 30%, 20% and 5% light irradiances during one day in June 2012 in Lin’an, China.

*A. roxburghii* plants were obtained from Lin’an commercial plantations and were maintained in a greenhouse at the Baicaoyuan test site of Zhejiang A & F University, China (30°15′N, 119°43′E). The photoperiod (day/night) and air relative humidity in the greenhouse were 14/10 h and 75% respectively. Plants were subjected to four different shade treatments for 40 days, beginning on June 1, 2012. Treatments consisted of 50%, 30%, 20%, or 5% natural irradiance, and were conducted in net houses (2.5 m high, 3 m long, and 2 m wide) covered with one or two layers of commercial plastic shading nets. Each treatment involved 10 pots with three replications (Figure 1). Diurnal variations of photosynthetically active radiation (400–700 nm wavelengths) were measured under all shade treatments with a TES-1332 digital lux meter (TES, Taiwan). Recorded data are displayed in Figure 2. All plants were kept well-irrigated and protected from bacterial pathogens and weed competition.

### Photosynthetic Parameters

Photosynthetic parameters were investigated using a LI-6400XT CO_2_/H_2_O porometer (Li-Cor, Lincoln, NE, USA). The parameters measured were net photosynthetic rate (P_n_, µmol m^−2^ s^−1^), stomatal conductance (G_s_, mmol m^−2^ s^−1^), and intercellular CO_2_ concentration (C_i_, µmol mol^−1^). During the treatment period, data were recorded between 9∶30 and 11∶30 am on days 0, 10, 20, 30, and 40. Air cuvette irradiance, temperature, and CO_2_ concentration were maintained at 1000 µmol m^−2^ s^−1^, 30°C and 380 µmol l^−1^ respectively, and the assimilation was recorded following a 10 min acclimation period [5]. At each conducted time point, five representative plants were randomly selected from each treatment and analyzed for the above parameters.

### Chlorophyll Fluorescence

Chlorophyll fluorescence of the same leaves used for determination of photosynthetic parameters was measured with a MINIPAM fluorometer (Walz, Effeltrich, Germany). Leaves were light-adapted for approximately 15 min prior to measurements of effective quantum yield of photochemical energy conversion (Yield) and photochemical (qP) and nonphotochemical (NPQ) quenching of chlorophyll fluorescence. The effective quantum yield of photochemical energy conversion at steady-state photosynthesis was calculated as Yield = (F_m_′ − F_s_)/F_m_′, where F_s_ and F_m_′ were fluorescence at steady-state photosynthesis and maximum fluorescence in the light, respectively. qP was calculated as (F_m_ − F_m_′)/(F_m_′ − F_0_), and NPQ was calculated as (F_m_ − F_m_′)/F_m_′ [32]. The relative rate of electron transport through PSII (ETR) was calculated as Yield × photosynthetically active radiation × 0.84 × 0.5 [33].

### Chlorophyll Content

Following measurement of chlorophyll fluorescence as described above, leaves were collected for determination of chlorophyll content (Chl a, Chl b, and Chl a+b). Chlorophyll pigments were extracted by grinding leaves in 80% acetone in the dark at room temperature, with their concentrations expressed as mg g^−1^ FW based on the equations of Porra [34].

### Chloroplast Ultrastructure

To examine chloroplast ultrastructure of mesophyll cells, sampled leaves described above were immediately fixed in 2.5% (v/v) glutaraldehyde (0.1 mol l^−1^ phosphate buffer, pH 7.2) for at least 48 h after removal from plants. The samples were then immersed in 1% (v/v) osmium acid for post-fixation, resin embedding, and ultrathin sectioning for transmission electron microscopy (H7650, Hitachi, Tokyo, Japan).

### Physiological and Biochemical Assays

Approximately 0.5 g leaf samples were collected on treatment days 0, 10, 20, 30, and 40 frozen immediately at −80°C. Protein content was determined based on the Bradford method [35], with bovine serum albumin employed as a standard. Crude enzyme preparation for superoxide dismutase (SOD) and peroxidase (POD) assays involved tissue homogenization in 5 ml of 100 mM potassium phosphate buffer (pH 7.0) following the procedure of He et al. [36]. Assays used for POD, SOD, and catalase (CAT) were those described by Argandona et al. [37] and Yin et al. [38]. Soluble sugar (SS) content was determined using anthrone colorimetry as described by Li [39], and malondialdehyde (MDA) content was measured by the thiobarbituric acid method according to Deng et al. [40].

### Statistical Analysis

One-way analysis of variance (ANOVA) was carried out using SPSS software version 16.0 (SPSS, Chicago, IL, USA). Duncan’s multiple range test was employed to detect differences between means (with *P* set to 0.05).

## Results

### Photosynthesis

**Figure 3:**
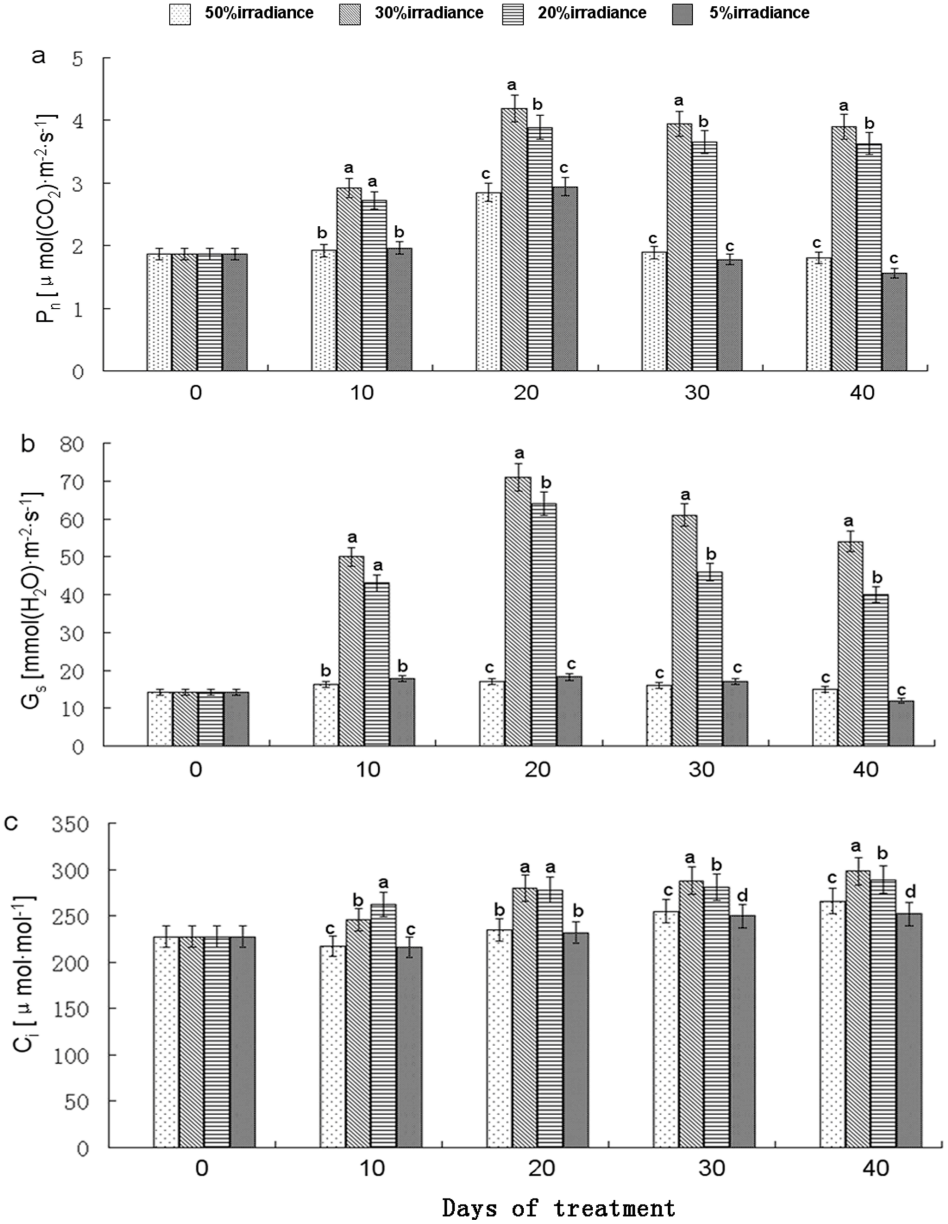
Net photosynthetic rate (P_n_) (a), stomatal conductance (G_s_) (b) and intercellular CO_2_ concentration (C_i_) (c). The values represented mean ± SE, and different letters mark significant differences among shade treatments on the same day (P<0.05).

P_n_ values of *A. roxburghii* varied significantly (*P*<0.05) among light intensities and treatment periods (Figure 3a). The P_n_ value was always highest at 30% irradiance, followed (in descending order) at most time points by 20%, 5%, and 50% irradiance treatments. P_n_ values significantly increased during the first 20 days of treatment, with the highest P_n_ value measured at 30% irradiance on day 20. Subtle changes were observed within treatments between days 30 and 40, but P_n_ values under 50% irradiance were always higher than those under 5% irradiance.

G_s_ values varied significantly among various light levels and exposure periods (Figure 3b). G_s_ values of plants subjected to 50% and 5% irradiance were always lower than those of 30%- and 20%-irradiance treated plants; on days 10 to 30, 50%-irradiance treated plants always exhibited the lowest values. The highest G_s_ values under 30%, 20%, and 5% irradiance were observed on the 20th day of treatment.

C_i_ values displayed slight increases over time (Figure 3c). On treatment day 10, C_i_ values from the 20% irradiance treatment were higher than those from other treatments. The highest values, however, were observed under 30% irradiance on days 20–40, and were higher than values measured during other treatments. Values measured from plants under 5% irradiance treatment were always the lowest.

### Chlorophyll Fluorescence

**Figure 4:**
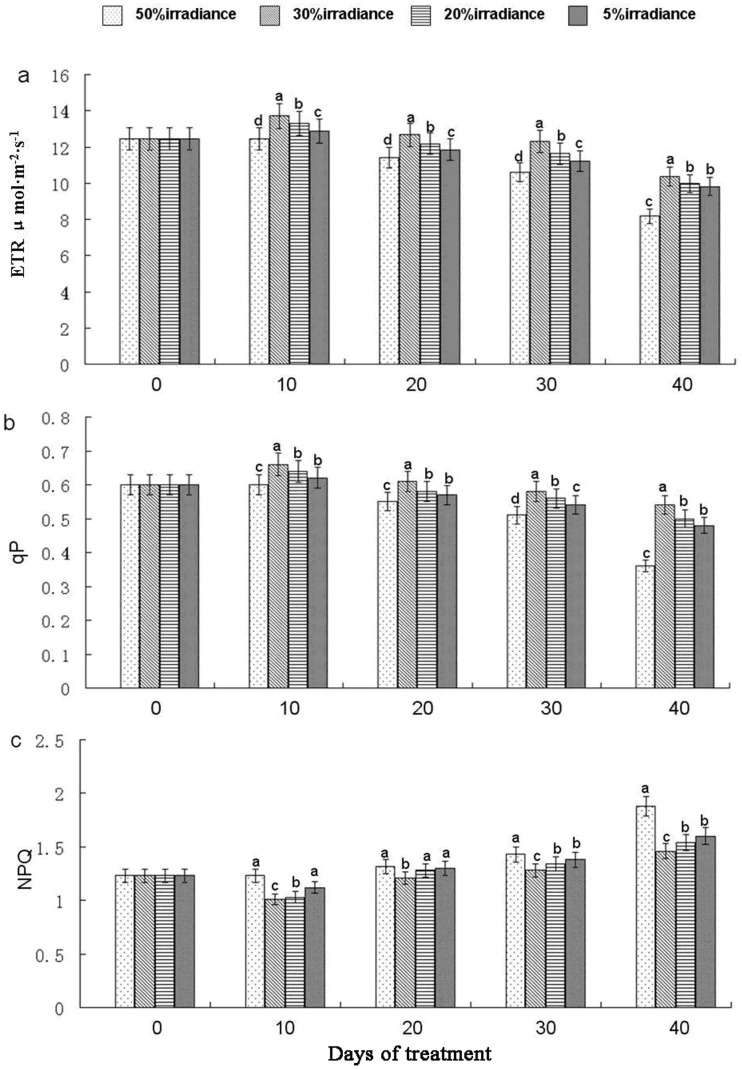
Electron transport rate (ETR) (a), photochemical quenching (qP) (b) and nonphotochemical quenching (NPQ) (c). The values represented mean ± SE, and different letters mark significant differences among shade treatments on the same day (P<0.05).

The 50% irradiance treatment resulted in a significant (*P*<0.05) reduction in apparent ETR and qP, and an increase in NPQ on the 40th day of treatment (Figure 4a–c). On day 10, the highest ETR value was recorded in leaves under 30% irradiance, while the lowest was found in plants grown under 50% irradiance. The trend observed for qP upon variation in light intensities and treatment times was similar to that of ETR. The highest and lowest qP values were observed in plants under 30% and 50% irradiance treatments, respectively. The highest NPQ values were always measured in 50% irradiance plants.

### Chlorophyll Contents

**Figure 5:**
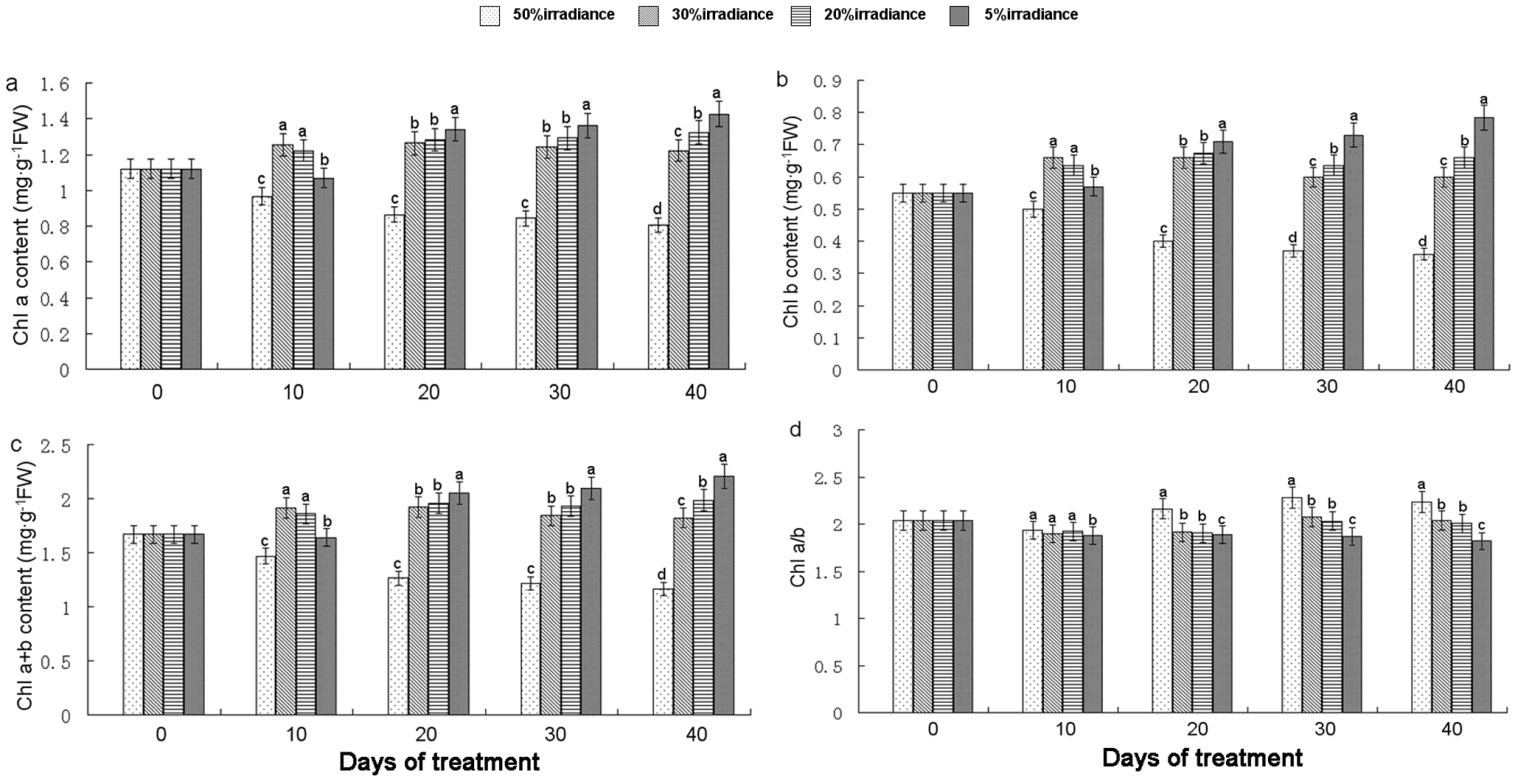
The Chl a content (a), Chl b content (b), Chl a+b content (c) and Chl a/b (d). The values represented mean ± SE, and different letters mark significant differences among shade treatments on the same day (P<0.05).

Chlorophyll content was significantly affected by different light intensities (Figure 5). Chl a and Chl b contents were increased and Chl a/b was decreased on days 20–40 of reduced irradiance treatments. The highest Chl a, Chl b, and Chl a+b contents were observed in 5%-treated plants on day 40. The shade treatments caused a decrease in Chl a/b over time, with the highest Chl a/b values observed in plants under 50% irradiance on day 40.

### Chloroplast Ultrastructure

**Figure 6:**
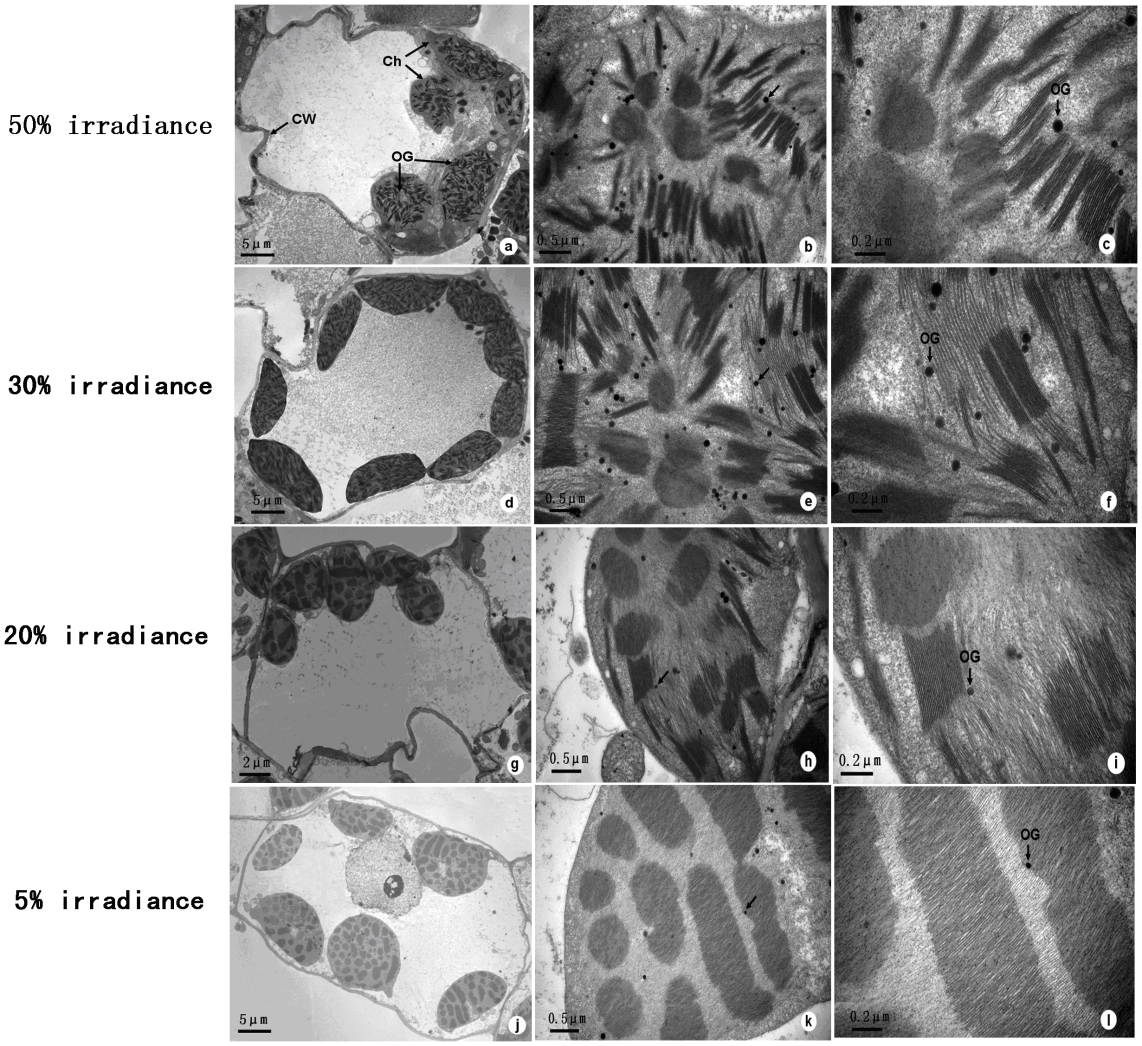
The chloroplast ultrastructure observed in the leaves of Anoectochilus roxburghii at 40 DOT. (a), (b), (c) the plants under 50% irradiance treatment; (d), (e), (f) the plants under 30% irradiance treatment; (g), (h), (i) the plants under 20% irradiance treatment; (j), (k), (l) the plants under 5% irradiance treatment. Notice the differences of the number of SG (indicated by arrow heads) and the number of grana lamella (indicated by arrows) between different irradiances. Abbreviation: Ch, chloroplast; CW, cell wall; OG, osmiophilic globules.

Chloroplast sizes and numbers were obviously influenced by light levels of *A. roxburghii* (Figure 6). The number of chloroplasts, grana, and grana lamellae generally increased as light irradiance was reduced. Most chloroplasts in leaves grown under 30% and 20% irradiance conditions exhibited normal ultrastructural organization, possessing a typical arrangement of grana and stroma thylakoids. Grana of plants grown under 30%, 20%, and 5% shading generally contained more thylakoids than those from plants grown under 50% irradiance. In addition, the number and size of osmiophilic globules was reduced in plants under 30% and 20% shading treatments compared with those from leaves grown under 50% and 5% treatments.

### Physiological and Biochemical Assays

**Figure 7:**
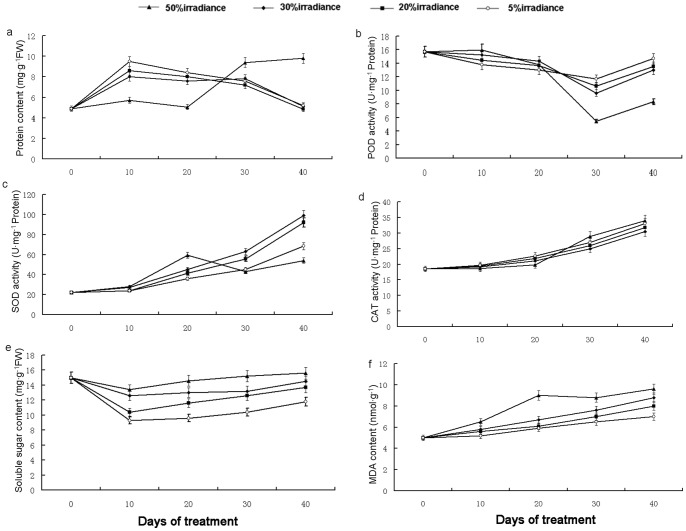
Protein content (a), POD activity (b), SOD activity (c), CAT activity (d), Soluble sugar content (e) and MDA content (f).

Under 50% irradiance conditions, leaf protein content remained relatively stable during the first 20 days of treatment, but increased rapidly thereafter. The other shaded plants reacted rather differently, however: during the first 10 days of treatment, leaf protein content increased, and subsequently declined (Figure 7a). A pronounced fall in leaf POD activity occurred after 20 days of treatment. On days 30–40, POD activity in 50% irradiance plants was significantly lower than in other shade-treated plants. Up to day 20, SOD activity in 50% irradiance plants was higher than in other shade-grown plants (Figure 5c). By day 40, relative SOD levels were reversed, with the highest expression (at 30% irradiance) being approximately double that of the 50% irradiance treatment. Shading had little influence on CAT activity up to the 20th day of treatment, but significant increases were observed thereafter. Up to day 20, 50% irradiance plants displayed the lowest CAT activity; on days 30–40, however, they exhibited the highest CAT activity levels. SS contents were positively correlated with the irradiance levels (Fig. 7e). SS content decreased at all levels of shade treated plants. Similarly, the response of MDA contents were also positively correlated with the irradiance levels. But its contents increased at all levels of treatments.

## Discussion

### Photosynthetic Response of *A. roxburghii* to Shading

C_i_ changed only slightly in *A. roxburghii* grown under different shade treatments, with the data suggesting that CO_2_ concentration was not the main factor reducing photosynthetic rate in leaves of plants under 50% and 5% irradiance treatments (Figure 3c). At the same time, values of G_s_ decreased significantly in plants grown under these treatments (Figure 3b, d). Under the high light environment, the observed reductions in G_s_ indicate that stomatal closure was due to light saturation and functioned to decrease water loss. When net CO_2_ assimilation became light saturated, transpiration constantly decreased with the declined photosynthetic photon flux density [11]. Thus under 5% irradiance treatment, plants also exhibit a similar behaviour as that in high light condition, that is, closing stomata due to water saturation to adapt to low light.

### Chlorophyll Fluorescence Response of *A. roxburghii* to Shading

Chlorophyll fluorescence measurement is a mainstay of studies of photosynthetic regulation and plant responses to the environment because of its sensitivity, convenience, and nonintrusive characteristics [12], [13]. ETR represents the relative quantity of electrons passing through PSII during steady-state photosynthesis [14]. Exposure to the high irradiance conditions of 50% irradiance resulted in a greatly reduction in ETR value (Fig. 4a). Reductions in ETR may be due to the loss of chlorophyll via and the reduction in the efficiency of excitation capture, which most likely as a result of photoinhibition [15]. qP is an indicator of the proportion of open PSII reaction centers [16]. A high qP is advantageous for the separation of electric charge in the reaction center, and is also beneficial to electron transport and PSII yield [17], [18]. In this experiment, observed differences in qP values revealed that *A. roxburghii* had significant differences in PSII electron transport activities when plants were grown under varied shade treatments. Electric charge separation in the reaction center, electron transport ability, and quantum yield of PSII were enhanced under 30% irradiance and weakened under 50% irradiance. NPQ is a reflection of the amount of unused energy from photosynthetic electron transport that is dissipated harmlessly as heat energy from PSII antennae [19], [20]. The low NPQ measured for 30% irradiance treatment plants indicates that these plants were able to effectively reduce irradiance heat and efficiently utilize the energy absorbed by PSII antenna pigments [17]. In contrast, the high NPQ observed in plants under 50% irradiance demonstrates that the energy absorbed in the physiologically usable range of light was much higher than the quantity photochemically usable, which would cause inhibition of photosynthetic capacity.

### Chlorophyll Content Response of *A. roxburghii* to Shading

Leaf chlorophyll content is an important determinant of photosynthetic rate [18] and dry matter production [21]. Naidu et al. [22] have suggested that reduced rates of photosynthesis may be due to reduced levels of chlorophyll, particularly Chl a, which is more directly involved in determining photosynthetic activity [23]. Decreases in Chl a and Chl b contents may thus reflect destruction of pigments by excessive irradiance. We observed significant (*P*<0.05) decreases in chlorophyll contents (Chl a, Chl b, and Chl a+b) under 50% irradiance conditions, suggesting that high irradiance may seriously impair the photosynthetic system. Plants grown under shaded conditions are known to optimize their light absorption efficiency by increasing pigment density per unit leaf area [24]. The reductions we observed in Chl a/b ratios in leaves of 30%, 20% and 5% irradiance plants were due primarily to significant (*P*<0.05) increases in Chl b content, most likely as a result of changes in the organization of both light harvesting and electron transport components [25]. The marked increase in leaf Chlorophyll content under 5% irradiance conditions demonstrates the ability of plants to maximize their light-harvesting capacity under low-light growth conditions [26].

### Chloroplast Ultrastructure Response of *A. roxburghii* to Shading

Leaves from 30%, 20%, and 5% shade treatments possessed grana containing more thylakoids than those of leaves grown under high (50%) irradiance conditions, and, as a consequence, had higher photosynthetic rates and pigment contents. Leaves grown in 30% irradiance environments exhibited better-developed chloroplasts, grana, and stroma lamellae. This result implies that 30% irradiance treatments were somewhat conducive to plant growth. Modulation of chloroplast development through increases in numbers of thylakoids, grana, and grana lamellae may be an important shade-tolerance mechanism in *A. roxburghii*. The number and size of osmiophilic globules can also be used as an indicator of photosynthetic efficiency [27], [28]. Chloroplasts in leaves of 30%- and 20%-treated plants contained the fewest and smallest osmiophilic globules; this result also suggests that moderate shading, to some extent, was beneficial, whereas 50% and 5% shade treatments were harmful to plant growth.

### Physiological and Biochemical Response of *A. roxburghii* to Shading

Performances of leaf protein content and antioxidant enzyme activities revealed that these traits were under strong genetic control, whereas SS and MDA contents were largely determined by the degree of shading. Exposure to high (50%) irradiance conditions greatly increased total protein content. Activity profiles of the various antioxidant enzymes were not uniform. After 40 days of shading, POD and SOD levels were significantly higher in 30%, 20%, and 5% irradiance plants than in 50% irradiance plants, whereas CAT activity remained lower. During these experiments, we carefully maintained consistent moisture availability and temperature conditions. Consequently, it is clear that POD, SOD, and CAT activity levels were not only markedly affected by the degree of imposed shading, but were also heavily influenced by the developmental status of the experimental plants. As SSs are an important carbon source and osmoregulator of plant growth, SS levels reflect plant nutritional status [29]. In our study, plant SS content was lowest under high irradiance treatments, and was responsive to shading severity. This result suggests that *A. roxburghii* responds differently to light with respect to carbohydrate metabolism. MDA content is considered to be an indicator of cellular membrane lipid peroxidation [30], [31]. The significantly high MDA content observed in our study under high irradiance indicates the occurrence of damage due to excessive irradiance.

## Conclusions

Our study, which measured photosynthetic characteristics associated with light stress sensitivity in *A. roxburghii*, has contributed to an understanding of this species’ shade tolerance. Our results demonstrate that shading is necessary for its normal growth, although different degrees and durations of shading treatments significantly influence photosynthetic activity, chlorophyll content, chlorophyll fluorescence, chloroplast ultrastructure, and physiological and biochemical indexes. Plants subjected to 50% irradiance conditions suffer photoinhibition because of excess light exposure, whereas those grown under 5% irradiance suffer from light deficiency. *A. roxburghii* adapts to shade conditions through increased levels of chloroplasts, grana, and grana lamellae, and higher POD and SOD activitities.
